# Comparison of bone mineral density according to domains of sedentary behavior in children and adolescents

**DOI:** 10.1186/s12887-022-03135-2

**Published:** 2022-02-01

**Authors:** Diego Giulliano Destro Christofaro, William Rodrigues Tebar, Bruna Thamyres Ciccotti Saraiva, Gabriela Caroline Rodrigues da Silva, Amanda Barbosa dos Santos, Gregore Iven Mielke, Raphael Mendes Ritti-Dias, Jorge Mota

**Affiliations:** 1 School of Technology and Sciences, Presidente Prudente, São Paulo State University (Unesp), Roberto Simonsen street, number 305, Presidente Prudente, Sao Paulo 19.060-900 Brazil; 2 School of Human Movement and Nutrition Sciences, The University of Queensland, Brisbane, Australia; 3 Universidade Nove de Julho (UNINOVE), Sao Paulo, Brazil; 4 Research Center on Physical Activity, Health and Leisure (CIAFEL), Faculty of Sport, University of Porto, Porto, Portugal

**Keywords:** Adolescent, Bone density, Child, Health, Sedentary

## Abstract

**Background:**

Somatic maturation and the age at onset of puberty are closely related to bone mineral density (BMD), and are potential confounders of the associations between physical activity, sedentary behavior (SB) and BMD in adolescents. Thus the aim was compare BMD at different anatomical sites according to different domains of SB.

**Methods:**

The sample consisted of 88 young people (54 boys and 34 girls; 9.5 ± 1.5 years). The self-reported SB was measured by the time spent on TV, computer, video game and smartphone. BMD at each location and throughout the body was assessed by DEXA. Physical activity was assessed by a questionnaire. The comparison of the different types of BMD sites according to the SB levels for each screen device and the total SB were analyzed by Covariance Analysis (ANCOVA).

**Results:**

Whole-body BMD was higher in young people with low total SB (Total BMD = 0.957 ± 0.042) than in those with moderate (Total BMD = 0.921 ± 0.053) and high SB (Total BMD = 0.929 ± 0.051) (*p*-value = 0.011). Children and adolescents with low total SB had higher BMD legs (0.965 ± 0.056) than young people with high total SB (BMD legs = 0.877 ± 0.209), but this relationship was attenuated when the analyzes were adjusted for physical activity (*p*-value = 0.068).

**Conclusion:**

Adolescents with high sedentary behavior tend to have lower whole body bone mineral density than those with low sedentary behavior.

## Introduction

The osteogenic effects of high impact and intense physical activities during childhood and adolescence has been considered an important determinant of bone mineral density (BMD) [1–5]. More recently, higher time spent in sedentary behavior (activities with an energy expenditure ≤1.5 metabolic equivalent of task in seated, reclined, or lying position [6]) has been associated with lower BMD in youth population [7–9]. Ivuškāns et al .[10], observed that increase in device-measured sedentary time over one year predicted negative changes in BMD of peripubertal boys independently of physical activity levels at light, moderate, and vigorous intensity.

In addition to the time spent in sedentary behaviors, the type/domain of sedentary behavior (e.g. TV time, computer, video game and cell-phone) is also relevant for several health parameters, including blood pressure and obesity [11–13]. However, the influence of sedentary behavior in bone health of children and adolescents is unclear among studies which used questionnaires, presenting different samples (only boys or girls), wide age range, as well as different sedentary behavior measurements [14]. While some studies report a negative association between screen-based sedentary behavior [8, 15], other findings report a positive relationship between BMD with internet use for non-scholar purposes and no association with television viewing, computer use, or playing videogames in adolescents [16].

Therefore, this study aimed to compare BMD at different body regions according to different levels of screen-based sedentary behaviors in children and adolescents, and investigate whether these differences vary by somatic maturation and physical activity levels.

## Methods

### Sample

This cross-sectional study was conducted in the Presidente Prudente, Brazil. The study was conducted throughout a partnership with a Philanthropic Institution, from which the young people were recruited. The Institution attends over 150 children and adolescents per year. Its aims are to ensure adequate care for children and adolescents of both sexes, especially essential protection, through services of institutional reception, coexistence, and strengthening of ties, along with the Brazilian Statute of the Child and Adolescent (ECA). The Institution receives funding from the government and donors. All protocols of the study were approved by the Ethics and Research Committee of the Institution responsible for this study.

Of 150 children who attend the Philanthropic Institution, the sample consisted of 88 children and adolescents (54 males and 34 females; [62 youth did not answer the questionnaire]) aged 6 to 14 years (9.5 ± 1.5). The following inclusion criteria were adopted: 1) aged between 6 and 14 years; 2) enrolled in the Philanthropic Institution; 3) presenting the Informed Consent Form signed by parents and/or guardians.

### Anthropometric measurements

Participants attended a clinic. Body mass was measured using a digital reading scale (Filizzola PL 150, model Filizzola Ltda, with a precision of 0.1 kg). Height and trunk length were measured using a stadiometer (Sanny, American Medical model of Ltda, with an accuracy of 1 mm). The length of legs was estimated considering the height and length of the trunk.

### Somatic maturation

Somatic maturation was estimated separately for boys and girls using the Maturity Offset estimation formula for young [17]. This calculates an overall score based on weight, height, trunk length and leg length, with negative scores indicating the remaining number of years for the individual to reach the peak of maturation, whereas positive scores represent the number of years a participant has passed the peak.

### Bone Mineral Density and Lean Mass

The dual-energy x-ray absorptiometry (DEXA, Lunar DPX-NT; General Electric Healthcare, Little Chalfont, Buckinghamshire, UK) technique was used to measure lean mass in kilograms (kg) and bone mineral density (BMD) in grams per square centimetres (g/cm^2^) of arms, legs, trunk, pelvis, spine and whole body. The software of the equipment itself (GE Medical System Lunar, version 4.7) was used. The analysis procedures were reported in a previous study by Saraiva et al .[18]. The precision of the machine in terms of coefficient of variation was tested in a pilot study with 30 volunteers not involved in this study and resulted in an error lower than 1%.

### Sedentary Behavior

The screen-based sedentary behavior was assessed by a questionnaire from Brazilian National School-Based Health Survey [19]. The reproducibility of this instrument presented an accuracy index from 55.0 to 96.2% for boys and from 56.4 to 90.4% for girls [20]. The participants reported time spent watching TV, using a computer, playing video games, and on mobile phones during weekdays and weekends. A total SB score was generated as the sum of time spent on each of these domains, and was divided into tertiles for categorization as low (1st tertitle), moderate (2nd tertile) and high SB (3rd tertile). The use of tertiles was adopted due to have good agreement against objectively-measured sedentary time (Cohen’s κ-coefficients >0.7) [21].

### Physical activity

The habitual physical activity level was measured using the questionnaire proposed by Baecke et al .[22]. This instrument consisted of 16 questions, with scores ranging from one to five, divided into three specific domains: physical activity at school, sports practice, and leisure-time physical activity. According to specific formula, a dimensionless score was generated for each assessed domain and the sum of these scores corresponded to the total physical activity score. The Baecke questionnaire was validated against gold standard methods as doubly-labelled water [23] and validated for Brazilian adolescents, presenting good reproducibility (intraclass correlation coefficient from 0.69 to 0.82 for boys and from 0.55 to 0.85 for girls) [24].

### Statistical analysis

Sample characteristics were presented as mean and standard deviation. The comparison between different types of BMD sites according to tertiles of time on TV, computer, video game or cell-phone, and the total sedentary behavior were analyzed by Covariance Analysis (ANCOVA). Initially, gender, age, somatic maturation and lean mass were included as adjustment variables. Subsequently, physical activity was inserted to verify if the results of the comparisons would change (what happened in the comparison of the BMD of legs, in which the comparison difference was significant between the total sedentary behavior levels and ceased to exist after the insertion of the activity. physical [result shown in Table 6]). The effect size (ES) was calculated by Eta- Squared and the magnitudes of the differences were considered trivial (0.01-0.06); moderate (0.07-0.13) and large (≥0.14) according to the recommendations of Maher et al .[25]. The significance level adopted was 5% and the confidence interval considered was 95%. The statistical package used was the SPSS.

## Results

Table 1 shows the sample characteristics. Television and cell phone were the domains where the participants spent more time per day. The average total sedentary behavior of children and adolescents participating in this study can be considered high, as it was higher than 8 h per day.

**Table 1:** Sample Characterization

Variables	Mean	Standard deviation
Age (years)	9.52	1.56
Body Mass (kg)	39.2	12.64
Stature (cm)	141.61	10.01
Maturity Offset (years)	−4.32	0.97
Lean mass (kg)	25.79	5.67
Physical activity (Baecke’s score)	8.76	1.95
Arms BMD (g/cm^2^)	0.630	0.041
Legs BMD (g/cm^2^)	0.929	0.129
Trunk BMD (g/cm^2^)	0.753	0,067
Pelvis BMD (g/cm^2^)	0.890	0.111
Spine BMD (g/cm^2^)	0.811	0.141
Total BMD (g/cm^2^)	0.931	0.050
Television (h/day)	3.54	2.29
Computer (h/day)	1.65	0.90
Videogame (h/day)	1.32	0.23
Cell-phone (h/day)	3.95	2.66
Total sedentary behaviours (h/day)	8.96	5.59

BMD Bone mineral density

Physical activity was not associated with somatic maturation (*r* = 0.04; *p*-value = 0.698). However, relationships between somatic maturation and BMD were observed in arms (*r* = 0.33; *p*-value = 0.002); legs (*r* = 0.44; *p*-value <0.001); trunk (*r* = 0.40; *p*-value <0.001); pelvis (*r* = 0.40; *p*-value <0.001); spine (*r* = 0.31; *p*-value = 0.003) and whole body (*r* = 0.33; *p*-value = 0.002).

Comparisons between TV-time and computer use by different BMD sites are presented in Tables 2 and 3, respectively. Overall, there was no variation in BMD according to tertiles of TV-time and computer use.

**Table 2:** Comparison of BMD sites according TV use in children and adolescents

	Low SB (≤2 h)		Moderate SB (2.01-4.66 h)		High SB (≥4.67 hous)			
	Mean	SD	Mean	SD	Mean	SD	***P***-value	ES
Arms BMD (g/cm^2^)	0.623	0.043	0.628	0.041	0.639	0.038	0.051	0.076
Legs BMD (g/cm^2^)	0.929	0.090	0.955	0.094	0.903	0.182	0.493	0.012
Trunk BMD (g/cm^2^)	0.750	0.076	0.760	0.070	0.748	0.056	0.635	0.011
Pelvis BMD (g/cm^2^)	0.895	0.139	0.901	0.104	0.873	0.084	0.956	0.001
Spine BMD (g/cm^2^)	0.818	0.208	0.812	0.095	0.802	0.082	0.920	0.000
Total BMD (g/cm^2^)	0.927	0.051	0.939	0.053	0.929	0.049	0.504	0.017

*TV* Television, *SB* Sedentary behavior, *SD* Standard deviation, *ES* Effect size, *BMD* Bone mineral density Adjusted by sex. age. maturity offset. lean mass and physical activity

**Table 3:** Comparison of BMD sites according computer use in children and adolescents

	Low SB (0 h)		Moderate SB (0.01-1.99 h)		High SB (≥ 2 h)			
	Mean	SD	Mean	SD	Mean	SD	***P***-value	ES
Arms BMD (g/cm^2^)	0.635	0.041	0.629	0.039	0.627	0.043	0.369	0.024
Legs BMD (g/cm^2^)	0.929	0.088	0.942	0.099	0.916	0.181	0.373	0.024
Trunk BMD (g/cm^2^)	0.753	0.065	0.747	0.071	0.757	0.069	0.641	0.011
Pelvis BMD (g/cm^2^)	0.883	0.103	0.887	0.120	0.898	0.115	0.765	0.007
Spine BMD (g/cm^2^)	0.804	0.090	0.784	0.090	0.841	0.206	0.707	0.009
Total BMD (g/cm^2^)	0.930	0.055	0.931	0.052	0.933	0.046	0.929	0.002

*SB* Sedentary behavior, *SD* Standard deviation, *ES* Effect size, *BMD* Bone mineral density Adjusted by sex. age. maturity offset. lean mass and physical activity

Table 4 shows that those with low video game time had higher leg BMD than participants with higher time spent in video game and that the magnitude of this difference can be considered moderate. (ES = 0.104). Children and adolescents with high sedentary behavior in video games had higher BMD values than those with moderate behavior (ES = 0.801).

**Table 4:** Comparison of BMD sites according videogame use in children and adolescents

	Low SB (0 h)		Moderate SB (0.01-1.16 h)		High SB (≥ 1.17 h)			
	Mean	SD	Mean	SD	Mean	SD	***P***-value	ES
Arms BMD (g/cm^2^)	0.632	0.040	0.626	0.045	0.624	0.040	0.468	0.019
Legs BMD (g/cm^2^)	**0.954**	**0.095**	**0.906**	**0.081**	**0.826**^**a**^	**0.270**	**0.013**	**0.104**
Trunk BMD (g/cm^2^)	0.761	0.072	0.736	0.055	0.733	0.052	0.469	0.019
Pelvis BMD (g/cm^2^)	0.901	0.118	0.872	0.104	0.853	0.081	0.859	0.004
Spine BMD (g/cm^2^)	**0.814**	**0.089**	**0.762**	**0.067**	**0.884**^**b**^	**0.349**	**0.035**	**0.081**
Total BMD (g/cm^2^)	0.936	0.051	0.926	0.052	0.917	0.048	0.483	0.018

*SB* Sedentary behavior, *SD* Standard deviation, *ES* Effect size, *BMD* Bone mineral density Adjusted by sex. age. maturity offset. lean mass and physical activity.;

^a^ Statistically significant when compared to low SB;

^b^ Statistically significant when compared to moderate SB

No significant differences were observed when compared to BMD at different sites according to sedentary behavior levels using cell-phone as presented in Table 5.

**Table 5:** Comparison of BMD sites according cell-phone use in children and adolescents

	Low SB (≤1.5 h)		Moderate SB (1.51-4.65 h)		High SB (≥4.66 h)			
	Mean	SD	Mean	SD	Mean	SD	***P***-value	ES
Arms BMD (g/cm^2^)	0.627	0.035	0.637	0.044	0.619	0.042	0.724	0.008
Legs BMD (g/cm^2^)	0.911	0.069	0.947	0.169	0.918	0.103	0.836	0.004
Trunk BMD (g/cm^2^)	0.741	0.061	0.765	0.068	0.743	0.077	0.834	0.005
Pelvis BMD (g/cm^2^)	0.871	0.106	0.903	0.106	0.891	0.135	0.926	0.002
Column BMD (g/cm^2^)	0.783	0.075	0.842	0.187	0.785	0.090	0.921	0.023
Total BMD (g/cm^2^)	0.920	0.048	0.943	0.049	0.924	0.054	0.491	0.017

*SB* Sedentary behavior, *SD* Standard deviation, *ES* Effect size, *BMD* Bone mineral density Adjusted by sex. age. maturity offset. lean mass and physical activity

Table 6 presents information on comparisons of the different BMD sites considering total sedentary behavior levels. It was observed in a preliminary model (without adjustment for physical activity) that children and adolescents with low sedentary behavior had higher leg BMD than those with high sedentary behavior (p-value = 0.027; ES = 0.085). However, when analyses were adjusted for physical activity, this comparison was no longer statistically significant. Children and adolescents with low sedentary behavior had higher whole-body BMD values than young people with moderate and high sedentary behavior.

**Table 6:** Comparison of BMD sites according total sedentary behaviors in children and adolescents

	Low SB (≤6.67 h)		Moderate SB (6.68-8.99 h)		High SB (≥9 h)			
	Mean	SD	Mean	SD	Mean	SD	***P***-value	ES
Arms BMD (g/cm^2^)	0.641	0.041	0.624	0.041	0.632	0.039	0.202	0.039
Legs BMD (g/cm^2^)*	**0.965**	**0.086**	**0.934**	**0.096**	**0.877**	**0.209**	**0.068**	**0.066**
Trunk BMD (g/cm^2^)	0.774	0.066	0.744	0.073	0.750	0.050	0.222	0.037
Pelvis BMD (g/cm^2^)	0.922	0.120	0.878	0.117	0.882	0.081	0.291	0.030
Column BMD (g/cm^2^)	0.830	0.084	0.786	0.088	0.849	0.255	0.207	0.038
Total BMD (g/cm^2^)	**0.957**	**0.042**	**0.921**^**a**^	**0.053**	**0.929**^**a**^	**0.051**	**0.011**	**0.174**

*SB* Sedentary behavior, *SD* Standard deviation, *ES* Effect size, *BMD* Bone mineral density Adjusted by sex. age. maturity offset. lean mass and physical activity;.; ^a^ statistically significant when compared to low SB. *This comparison lost significance after the insertion of physical activity as an adjustment variable

## Discussion

Our findings suggest that leg BMD was higher in children and adolescents with low sedentary behavior for video games than children with high SB; spine BMD was higher in children and adolescents with high SB in videogames than those with moderate SB for this type of electronic device. Moreover, those classified with low SB had higher leg BMD than adolescents with high SB, but these differences disappear after the insertion of physical activity as an adjustment variable. In whole body BMD, children and adolescents with low total SB had higher BMD when compared to those with high behavior, can indicates children’s physical fitness is potentially influenced by biological, behavioural and environmental factors [26].

Similar to our findings, Shao et al .[27] in a study of Chinese adolescents, found an inverse relationship between longer time playing video games and BMD from different body regions, such as the legs. One of the possible mechanisms to explain these results is that the shorter the time involved in playing video games, the more time available for physical activity, since these types of behavior have been negatively related in both overall activity and moderate-to-vigorous physical activity [28]. Therefore, lower physical activity levels may impact in BMD of legs due to the reduction in the osteogenic function of activity [29], which impacts on bone matrix [30]. Tebar et al .[4] observed a positive relationship between physical activity and BMD of legs among boys. Besides, high level of sitting time was negatively associated with bone strength in legs independently of moderate-to-vigorous physical activity [31], which also contributes for low BMD in adolescents with elevated time in screen-based sedentary behaviors as a cluster of exposure (low levels of moderate-to-vigorous physical activity and high levels of sedentary behavior).

Adolescents with high SB for video games had higher spine BMD when compared to adolescents with moderate behavior for video games. This finding is opposed to observed by Shao et al .[27] in Chinese adolescents, where the duration of playing videogames was negatively related to spine BMD, as well as lower legs, trunk, pelvic, and total BMD. One of the possible hypotheses for these findings is that in order to play video games children and adolescents have to spend considerable time sitting, but with the spine erect, thus working the back muscles in an isometric form, providing a mechanical load by isometric muscle contraction in this region for prolonged time of the which could contribute to increases in spine BMD, given the close relationship between muscle mass and BMD [32].

No significant differences in BMD from different body regions considering SB levels for TV, computer and smartphone were observed in our study. Previous studies have shown significant relationships between these screen devices and BMD. Whinter et al .[15] observed that high computer use was inversely related to BMD in Norwegian adolescents. Ivuškāns et al. [10] in a study with male adolescents from Estonia, they also observed relationships between high SB and BMD. The lack of associations in the present study might be explained by the characteristic of the sample, which includes Brazilian children and adolescents in social vulnerability, which may have low access to the use of computers and smartphones. Another reason may be due to differences in the forms of sedentary behavior measurement, since in the present study it was performed by questionnaire, against objectively measurements in the study by Ivuškāns et al .[10]. It also points out that our study considered adolescents of both sexes, while Ivuškāns et al .[10] assessed only boys.

In the analysis of total SB, a significant difference was observed between the BMD of legs in children and adolescents with low total SB than those youths that had high SB. However, when physical activity was added to the model, these comparisons lost their significance [8] observed that the participation of 3 h per week on extracurricular sports contributed to mitigate the effects of SB on girls’ bone health. In addition to the osteogenic effects of physical activity, another factor to consider is that many sports in Brazil are practised outdoors, where there is sun exposure and may have a higher vitamin D increase that is associated with better bone health [33]. However, these findings need further investigation. Constantini et al .[34] observed a positive relationship between physical activity and BMD, even in adolescents with vitamin D deficiency.

Children and adolescents with lower total SB had higher whole-body BMD values. High levels of screen-based SB has been associated with poorer sleep quality [35], and it is suggested that duration of sleep for less than 8 h could impair the bone mass accumulation in stages of fast growth, as adolescence [36]. Furthermore, long time SB could influence bone resorption [37]. In this sense, it is possible that the relationship between screen-based sedentary behavior and BMD in Brazilian children and adolescents could occur mainly in overall amounts, as an accumulation of SB occurs by different devices, which takes place of another daily activity of higher mechanical load, even of light intensities, and could result in an impairment of bone mass acquisition in fast growth stages, reflecting in poor bone health in adulthood [38].

As limitations of the present study we can mention the absence of eating habits, especially considering intake of vitamin D, calcium, and carbonated drinks. Another important aspect is that it was not possible to distinguish whether in the use of videogames, this activity was done actively (active-videogame) or by console, which should be considered a bias of this study. As strong aspects we can highlight the evaluation of different types of sedentary behavior and different body sities, considering the specificity of each domain. Another factor was the insertion of maturation as an adjustment variable, reducing the chances of possible bias in the analysis due to the strength of this variable with BMD in pediatric populations. Finally, it is emphasized that this study was developed in Brazil and that most of the data presented in the literature are from populations of European countries or North America, thus providing information from a South America’s low-middle income country.

As practical applications, the present study findings reinforce the need for strategies focused on reducing total screen-based sedentary behavior beyond increasing the practice of physical activity in children and adolescents. This actions could prevent an early lower bone mineral density in this population, providing a reduction in the risk of developing osteoporosis in the future.

## Conclusion

Adolescents with high sedentary behavior tend to have lower whole body BMD than those with low sedentary behavior. Health promotion actions should focus on transforming this type of behavior into more active activities in pediatric populations.

## Data Availability

The dataset of the study can be made available considering a justifiable request by contacting the first author.

## Abbreviations

BMD: Bone mineral density
SB: Sedentary behaviour
TV: Television
